# Supplementary material for: Long-Range Allosteric Communication Modulated by Active Site Mn(II) Coordination Drives Catalysis in Xanthobacter autotrophicus Acetone Carboxylase
Source: Int J Mol Sci. 2025 Jun 20;26(13):5945. doi: 10.3390/ijms26135945 (PMC12250083; doi:10.3390/ijms26135945)
Supplement: Supplementary file 1 [file ijms-26-05945-s001.zip › ijms-3689187-supplementary.pdf]

SUPPLEMENTAL INFORMATION

Long-range allosteric communication modulated by active site Mn(II) coordination drives catalysis in *Xanthobacter autotrophicus* acetone carboxylase

Jenna R. Mattice[1], Krista A. Shisler[2], Jadyn. R. Malone[1], Nic A. Murray[1], Monika Tokmina-Lukaszewska[1], Arnab K. Nath[1], Tamara Flusche[3], Florence Mus[3], Jennifer L. DuBois[1], John W. Peters[3*], and Brian Bothner[1*]

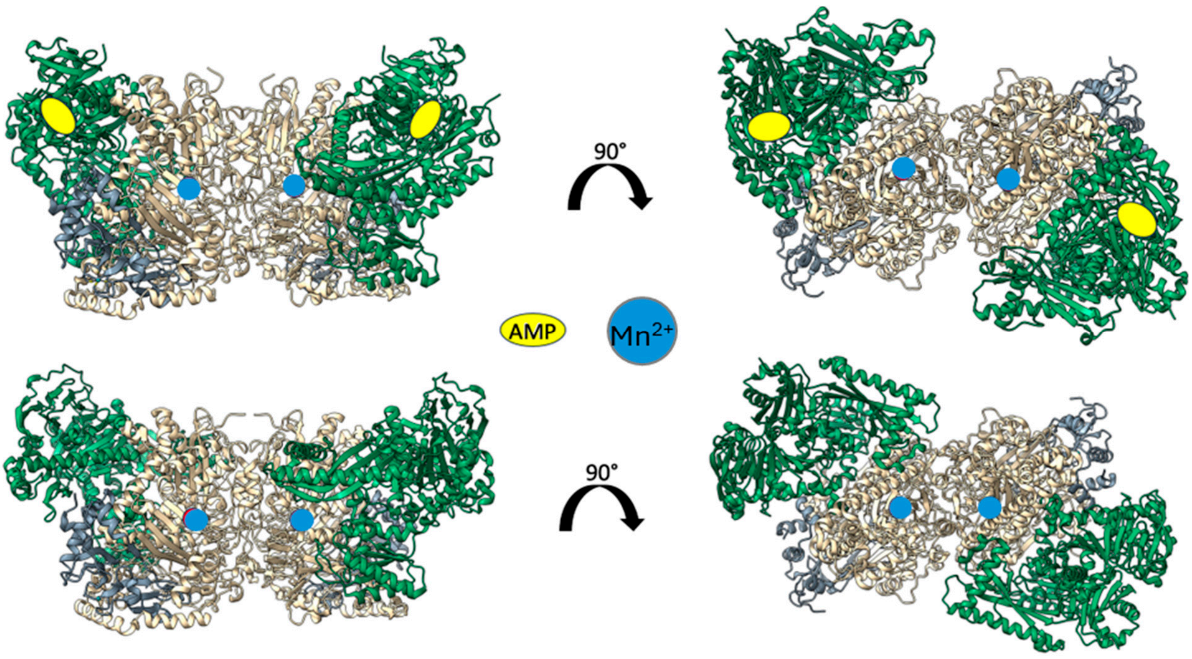

Figure S1. Structural model of XaAC. The beta subunit is in green, alpha subunit in tan and gamma in grey. Front and top view of α2β2γ2 heterohexamer. Top: AMP bound XaAC, PBD ID: 5SVB; bottom: ligand free XaAC, PDB ID: 5SVC. Total molecular weight of XaAC is ~ 360 kDa.

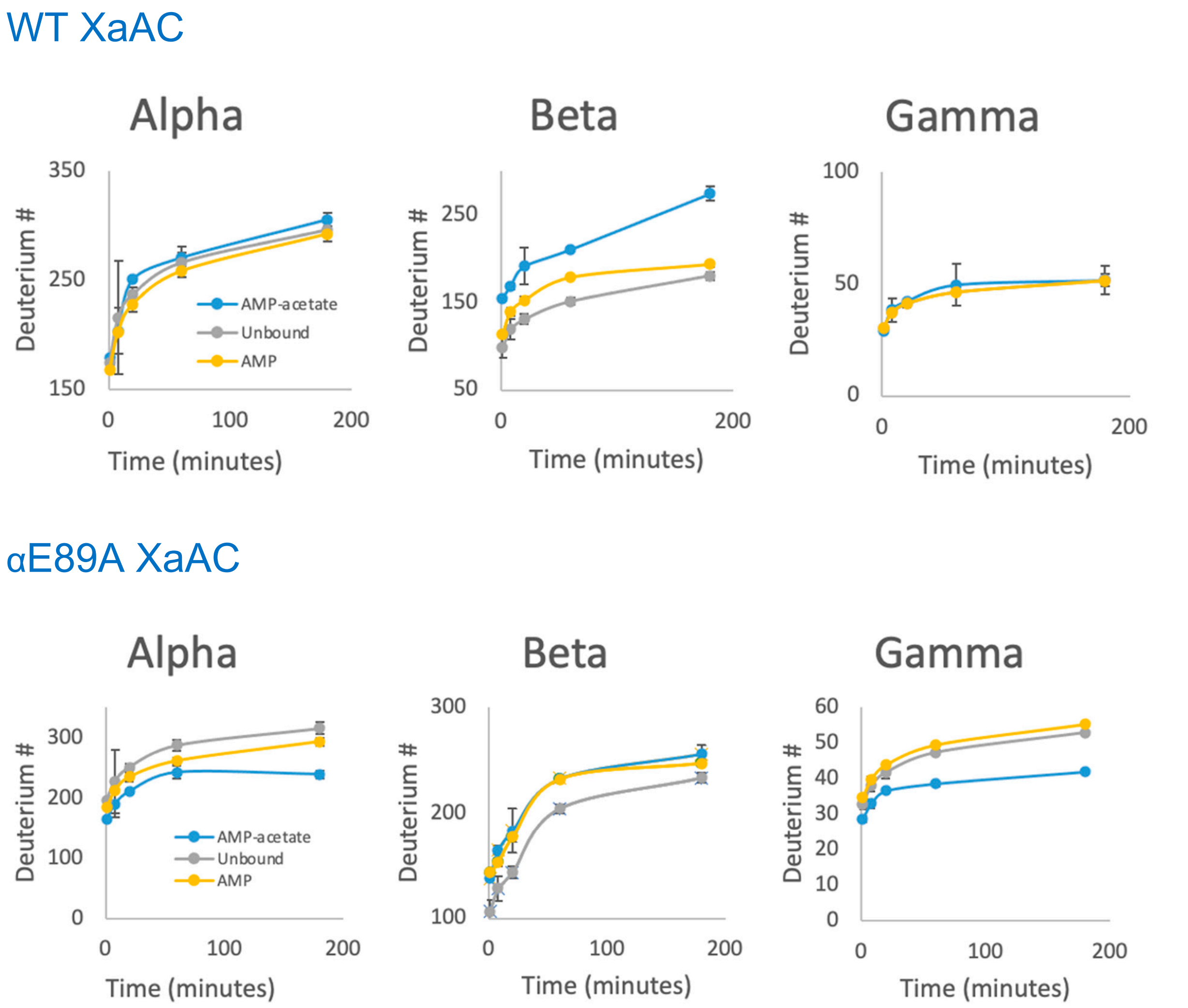

Figure S2. Intact protein HDX-MS highlights the impact of ligand binding on the hydrogen bonding network in AC. Top: Deuterium uptake curves over time shown for each subunit of WT XaAC. In the Beta subunit more uptake is seen in the AMP and AMP-acetate conditions compared to unbound. The alpha and gamma subunits do not have a global change in deuterium uptake upon substrate binding. The unbound condition in gamma is hidden behind the AMP condition. Error bars show standard deviation (n=3). Bottom: Deuterium uptake curves over time shown for each subunit of αE89A variant**.** The single amino acid substitution has a significant change on the global hydrogen bonding network with and without substrate. The alpha subunit shows the most exchange without substrate. The addition of AMP and AMP-acetate suppress exchange. In the beta subunit greater exchange was observed in the AMP and AMP-acetate conditions. The gamma subunit exchanged less with the addition of AMP-acetate. Neither alpha or gamma subunits changed in the WT AC

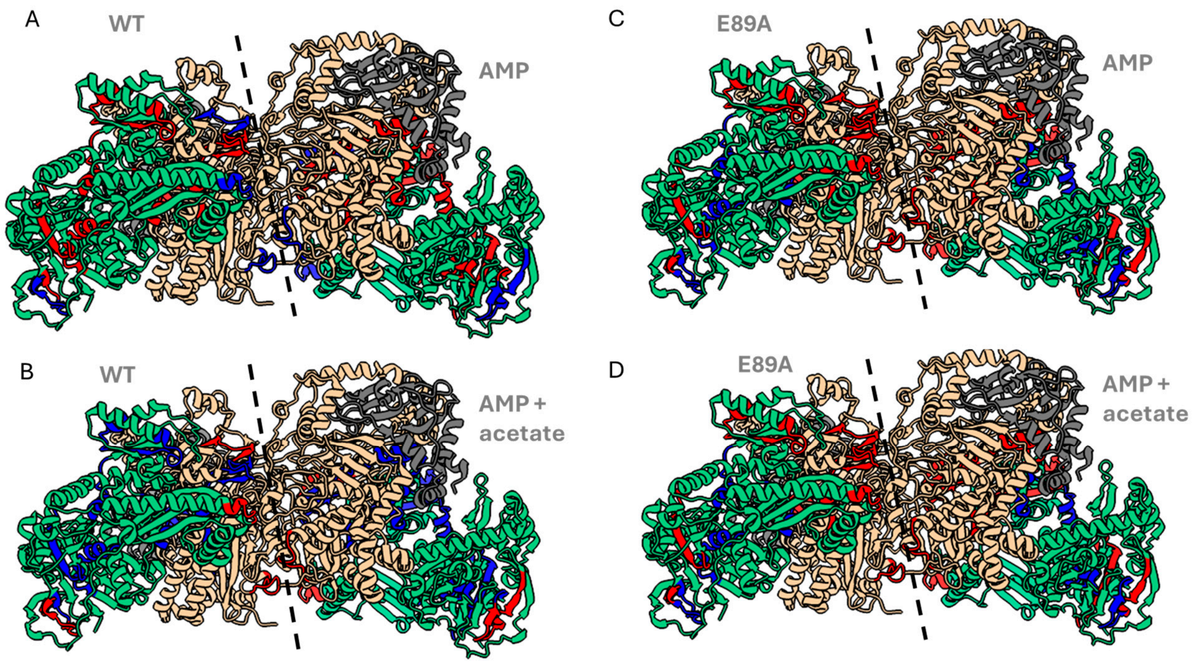

Figure S3. Deuterium uptake patterns of WT and αE89A XaAC shown as the heterohexamer. For perspective, peptides shown in Figures 4 are now shown on both halves of the complex. Red peptides have more exchange upon ligand binding, while blue peptides have less exchange upon ligand binding. A. Heterohexamer of WT AC comparing the exchange pattern of AMP bound with the unbound complex. The dashed line denotes the heterodimer interface. B. Heterohexamer of WT AC with exchange pattern of the unbound vs AMP-Acetate complex. C. Heterohexamer of αE89A AC with exchange pattern of the unbound vs AMP bound complex. D. Heterohexamer of αE89A AC with exchange pattern of unbound vs AMP-Acetate bound complex. αE89A shows greater dynamics at the 2-fold interface compared to WT.

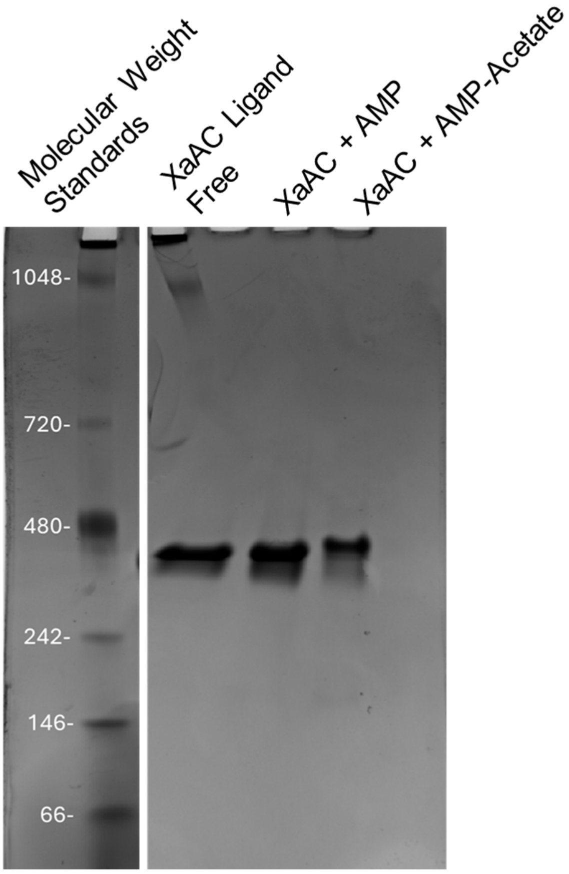

Figure S4. Native gel electrophoresis of Xa AC. Various purified forms of AC were purified by Size Exclusion Chromatography and then analyzed by native gel electrophoresis to confirm the protein complex remains intact and has the expected molecular mass. Mini-PROTEAN TGX Precast 4-15% gel from Bio-Rad. The running buffer was 192 mM glycine and 25 mM Tris. Gels was stained with Coomassie blue for visualization.

Table S1. Amino acid coverage of alpha, beta, and gamma subunits of XaAC.

	Xa AC Alpha Subunit Peptide Mapping	WT Xa AC Beta Subunit Peptide Mapping	WT Xa AC Gamma Subunit Peptide Mapping
# Amino Acids	747	682	151
Unique Peptides	244	214	47
% Coverage	95%	96%	85%

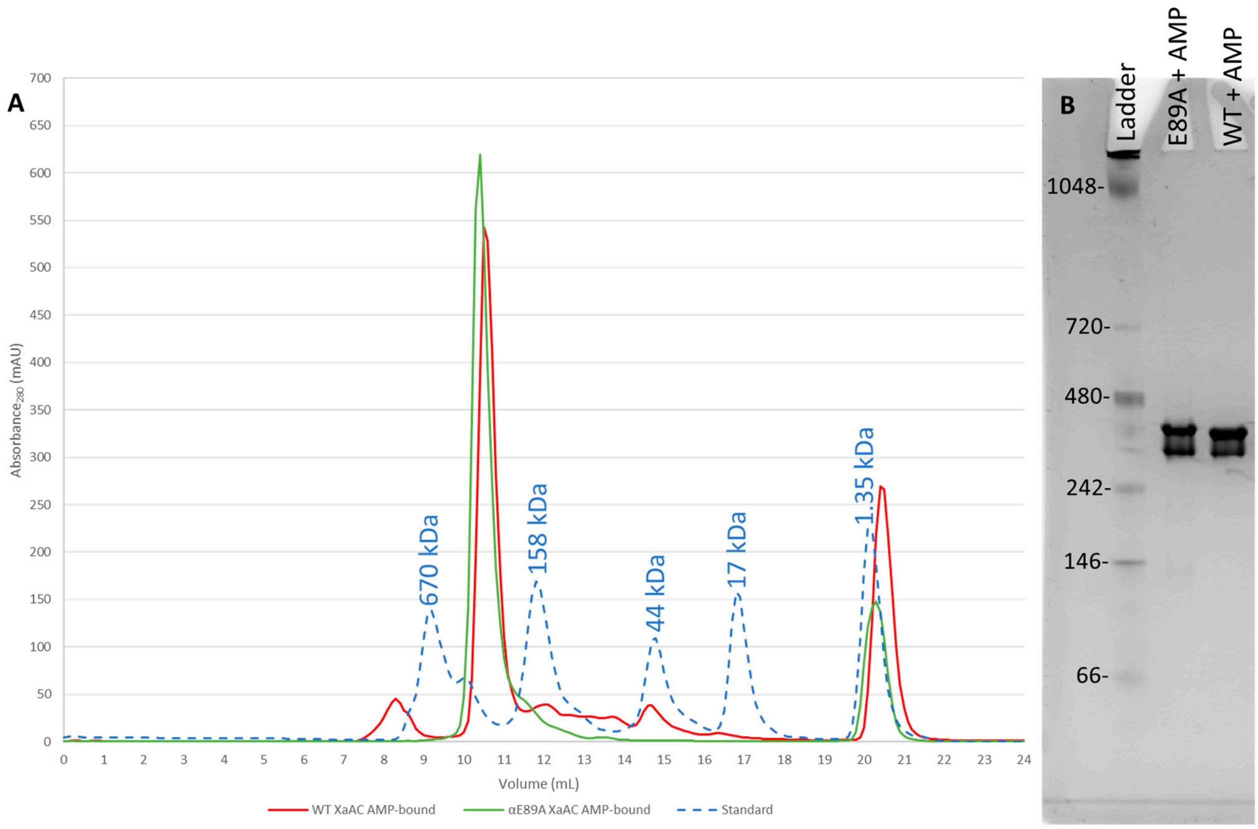

Figure S5. SEC and Native Gel Characterization of WT and αE89A XaAC
A) Retention times of WT and αE89A XaAC are the same during size exclusion chromatography. Purification profile of WT XaAC AMP-bound (red), αE89A XaAC AMP-bound (green), and Standard (blue, dashed) show that each protein predominately stays in the heterohexameric state. The peaks indicating AC complex elutes between the 670 kDa and 158kDa standards confirming a molecular weight of around 360 kDa. The peaks indicating unbound AMP elute with the 1.35 kDa standard as both are outside the fractionation range for the column used.

B) Native Gel Analysis of AMP-bound WT and αE89A XaAC. Both complexes run at the same size on Native-PAGE. Protein taken from the retention volume of 10.5 mL shows that AMP-bound WT and αE89A XaAC have the same estimated molecular weight at 360 kDa.
Methods: WT XaAC αE89A XaAC were incubated with 10 mM AMP and run on a Superdex 200 Increase 10/300 (Cytiva) in 50 mM Tris, pH 7.6, 150 mM NaCl, 10% glycerol. Fractions from the 10.5 mL elution peak were run on Native-PAGE using Mini-PROTEAN TGX Precast 4-15% gels from Bio-Rad. The running buffer used was 192 mM glycine and 25 mM Tris, and the gels were stained with Coomassie blue for visualization. The gel filtration standard (BioRad) was run according to the manufacturers instructions on the Superdex 200 Increase 10/300 (Cytiva).
